# Do task and item difficulty affect overestimation of one’s hand hygiene compliance? A cross-sectional survey of physicians and nurses in surgical clinics of six hospitals in Germany

**DOI:** 10.1186/s13756-022-01188-7

**Published:** 2022-12-02

**Authors:** Jonas Lamping, Ivonne Tomsic, Maike Stolz, Christian Krauth, Iris F. Chaberny, Thomas von Lengerke

**Affiliations:** 1 Department of Medical Psychology, Center of Public Health and Health Care, Hannover Medical School, Hannover, Germany; 2 Institute of Epidemiology, Social Medicine and Health Systems Research, Center of Public Health and Health Care, Hannover Medical School, Hannover, Germany; 3 Institute of Hygiene, Hospital Epidemiology and Environmental Medicine, Interdisciplinary Center for Infectious Medicine, Leipzig University Hospital, Leipzig, Germany

**Keywords:** Hand hygiene, Compliance, Overconfidence, Overestimation, “5 Moments of Hand Hygiene” (WHO-5), Physicians, Nurses, General/visceral surgery, Orthopedics/trauma surgery, Anesthesiology

## Abstract

**Background:**

One barrier to hand hygiene compliance is overestimation of one’s own performance. Overconfidence research shows that overestimation tends to be higher for difficult tasks, which suggests that the magnitude of overestimation also depends on how it is assessed. Thus, we tested the hypothesis that overestimation was stronger for hand hygiene indications with low compliance (i.e., high difficulty), and the hypothesis that self-reported overall compliance based on a single item is higher than based on “5 Moments of Hand Hygiene” (WHO-5) items, since the single item implies an aggregation across indications.

**Methods:**

In the WACH trial (German Clinical Trials Register [DRKS] ID: DRKS00015502), a questionnaire survey was conducted among physicians and nurses in nine surgical clinics (general/visceral surgery or orthopedics/trauma surgery) of six German hospitals. Self-reported compliance was assessed both by a single item and the WHO-5-items using percentage scales. These were compared with each other and with direct observations. Relative frequencies of the WHO-5 indications used to calculate the WHO-5-based self-reported overall compliance rate were estimated by a systematized review of the literature (see appendix). In analysis, t-tests, Chi^2^-tests and multiple linear regressions were used.

**Results:**

Ninety-three physicians (response rate: 28.4%) and 225 nurses (30.4%) participated. Significant compliance differences between physicians and nurses were found for direct observations and were in favor of nurses, while no such differences were found for self-reports. Across the WHO-5, overestimation showed inverse correlations with observed compliance (physicians: r = −0.88, *p* = 0.049; nurses: r = −0.81, *p* = 0.093). Support for the hypothesis that the self-reported overall compliance based on one item is higher than that based on WHO-5 items was found for physicians (M = 87.2 vs. 84.1%, *p* = 0.041; nurses: 84.4 vs. 85.5%, *p* = 0.296). Exploratory analyses showed that this effect was confined to orthopedic/trauma surgeons (89.9 vs. 81.7%, *p* = 0.006).

**Conclusion:**

Among physicians, results indicate stronger hand hygiene overestimation for low-compliance indications, and when measurements are based on a single item versus the five WHO-5 items. For practice, results contribute to infection prevention and control’s understanding of overestimation as a psychological mechanism that is relevant to professional hand hygiene.

**Supplementary Information:**

The online version contains supplementary material available at 10.1186/s13756-022-01188-7.

## Introduction

Overconfidence, defined as “greater confidence than reality justifies” [1, p. 1], has been termed “one of the most consistent, powerful and widespread” among psychological biases [2, p. 317] and the “rarely addressed mother of all biases” in medicine [3, p. 127]. To date, overconfidence in medicine has been primarily studied as a cause of diagnostic errors [4, 5], and one study found poor case management by overconfident health care workers [6]. As regards infection prevention and control (IPC), particularly hand hygiene, overconfidence has been argued to represent a barrier to professionals’ compliance with interventions to prevent health care-acquired infections (HAIs) in some but, given pertinent clinical experiences, surprisingly few studies [7–10]. Correspondingly, this applies to overestimation as well, i.e., assessing one’s actual performance to be better than it is. If health care workers genuinely believe that they disinfect their hands at 80% or more of the opportunities when it is indicated, they will perceive less need to change their behavior than if this subjective estimate is 50% or less – and will thus be farther away from optimal compliance with evidence-based guidelines.

To overcome this barrier, it is essential to obtain theoretical and empirical insight into the sources of such overconfident self-beliefs. In principle, motivational and cognitive factors can be distinguished from each other [11]. Among the former, desires to view and present oneself positively and/or better than others are the most-cited drivers of inflated self-assessments [11].

Among cognitive factors, the difficulty of the task on which one is judging one’s performance has been argued to be one of the key determinants that contribute to overestimation [12]. Specifically, people “… tend to overestimate their performance on hard tasks and underestimate it on easy tasks” [1, p. 3]. The mechanism behind this pattern is that given imperfect knowledge of one’s own true performance (which represents the usual case), subjective estimation of this performance will regress to the overall mean. This implies that for a difficult task, i.e., one with a relatively low average achievement rate, the subjective estimate will exceed this rate, while underestimation occurs in the case of easy tasks [13﻿]. On the level of operationalization, this pertains to items that assess subjective estimates as well, i.e., task difficulty translates to survey item difficulty [14].

When applying this reasoning to self-assessments of professional hand hygiene compliance, it is notable that in the aforementioned overconfidence studies [7–10], the surveyed health care workers were asked to estimate their *overall* hand hygiene compliance. That is, they were (implicitly) required to base their self-assessments on some form of aggregation across the five hand hygiene indications defined by the World Health Organization (WHO-5) [15]. This is comparatively difficult since these categories of hand hygiene opportunities—before patient contact, before an aseptic task, after body fluid exposure, after patient contact, and after contact with patient surroundings—vary not only with respect to compliance, but also in the frequency of their occurrence, i.e., the proportions of hand hygiene opportunities associated with each indication [16]. Thus, in the case of a single item respondents are required to apply some (at least implicit) weighting across the different indications. Correspondingly, the resulting estimate of self-reported compliance may have differed from one based on indication-specific items, since recalling distinct types of situations may be easier.

In addition, all of these studies [7–10] used Likert-scales with verbal cues such as “consequently” [7–9] or “never/always” [10] to indicate behavioral frequency and to evaluate subjective compliance. This introduced additional vagueness due to variation in individuals’ understanding of these terms. This is also true for three studies that, although focusing on different subjects, also reported [17, 20] or at least suggested [18] hand hygiene overestimation. Moreover, only four of the abovementioned studies provided compliance rates based on direct observations in their own study setting [10, 17, 18, 20]. In most of these studies, comparisons of observed compliance rates with the respective subjective estimates are flawed because these were reported either as the means of scores based on Likert scales [18] or as the percentages of respondents who reported at least “often” washing their hands [17], or using the highest values of a Likert-scale [10]. Thus, scaling across the variables that were compared is very different, and this critically challenges the validity of the inferred overestimation. The only studies we found in which a percentage scale was used for both observed and self-reported compliance found large overestimation for overall compliance in a Southeast Asian context (specifically, in Thailand: 82.4% vs. 23.2%) [19], while a recent study in three tertiary care hospitals in Germany found no overall overestimation (76.9% vs. 74.9%) [20]. Both studies included physicians and nurses, but did not report results stratified for these two professions.

Against this background, the present study analyzed data collected as part of a survey of health care workers in six teaching and two general hospitals in Germany. Specifically, physicians and nurses working in either surgery (general/visceral surgery or orthopedics/trauma surgery) or anesthesiology estimated, via a written questionnaire on the prevention of surgical site infections, their personal hand hygiene compliance (in %) both overall and, in a later section of the questionnaire, for each of the WHO-5 indications. In analyzing these self-reports, on the one hand we tested whether overestimation occurred in this sample of health care professionals as well by comparing their subjective compliance rates with the rates identified by direct observation in the participating hospitals. This was done for both overall and indication-specific compliance, the hypothesis being that overestimation was stronger for high difficulty, i.e., indications with low compliance. On the other hand, our main hypothesis related to the comparison of the self-reported overall compliance rate based on one item, termed the “single item overall compliance rate”, with the overall compliance rate calculated based on the five self-reported WHO-5-specific rates, termed the “WHO-5-based overall compliance rate”. To calculate this rate, weights for the relative occurrence identified for the five indications in a systematized review by the first author were used; see Methods-section and “Additional file 1” document. Specifically, our hypothesis is that the “single item overall compliance rate” would be significantly higher than the “WHO-5-based overall compliance rate”. The basis for this hypothesis was the higher degree of difficulty of the single item assessment, as delineated above. All analyses were stratified for physicians and nurses, since lower hand hygiene compliance has been found for physicians than for nurses [16, 21–24], and the identification of specific determinants of physicians’ hand hygiene behavior has been proposed as a key contemporary research topic in IPC [25].

## Methods

### Study design, setting and participants, and procedure

A cross-sectional survey using a self-administered written questionnaire was conducted from March to October 2019 within the baseline assessments of the WACH trial (“Wundinfektionen und Antibiotikaverbrauch in der Chirurgie” [“Wound Infections and Antibiotics Use in Surgery”]; German Clinical Trials Register-ID: DRKS00015502) [26]. All physicians and nurses working in either general and visceral surgery, orthopedic and trauma surgery, or the associated anesthesiology departments of the six hospitals participating in the trial were invited to take the survey. The six hospitals were deliberately recruited from outside the university sector and were chosen to make it possible to transfer behavioral approaches from a previous project in a tertiary care university hospital [27, 28]. Overall, there were nine surgical clinics (four general/visceral clinics and five orthopedic/trauma clinics), ranging in size from 50 to 196 beds (mean: 119, standard deviation: 56.4), 2,145 to 7,254 surgical procedures per annum (p. a.) (x̄: 4,074, SD: 2,023.1), 14,990 to 60,007 patient days p. a. (x̄: 31,191, SD: 16,267.5), and average duration of stay ranging from 6.1 to 11.3 days (x̄: 7.7, SD: 1.7). Sufficient copies of the questionnaire were provided to the IPC teams of each hospital by the WACH project center. Within each hospital, the questionnaires were distributed to the clinicians by the IPC team, and the members of that team were invited to seek supervision and support from the WACH team if necessary. Informed consent was appropriately obtained. Field time per hospital depended on hospital size, the number of participating clinics, and the working capacity of the local IPC team, and ranged from 14 to 97 days (x̄: 56, SD: 29.3). Overall, the questionnaire included 94 items dealing with the themes of awareness of and compliance with 26 SSI-preventive clinical interventions, the psychosocial determinants of this compliance, the respondents’ awareness of IPC implementation interventions, and sociodemographics. Information on the items and on the transformed variables relevant to the present analyses is provided in the following section. Information on the direct observation of hand hygiene compliance conducted within the participating hospitals of the WACH project is also provided. This information is used in the present context for comparison with the subjective estimates.

### Measures

#### Sociodemographic characteristics

In the questionnaire, respondents reported their sex and age; the latter was assessed in categories (< 18, 18–30, 31–40, 41–50, 51–60 and > 60 years) to comply with local data protection regulations. Classification of the respondents into four occupational groups (surgeons, anesthesiologists, surgical nurses, and anesthesiology nurses) was performed based on two questionnaire items that asked about the respondents’ departments (orthopedics/trauma surgery and general/visceral surgery) and specialties (surgical specialist, surgical resident, anesthesiology specialist, anesthesiology resident, physician assistant, ward nurse, perioperative nurse or anesthesiology nurse).

#### Self-reported hand hygiene compliance

For the *single-item overall compliance rate,* the following item was used: “Please estimate the proportion of opportunities in which you compliantly disinfect your hands, i.e., when indicated, (in %): __ %”. In a later part of the questionnaire, WHO-5 indication-specific compliance rates were assessed based on five items, as follows: “Please estimate the proportion of opportunities in which you disinfect your hands… …before patient contact, (in %): __%/ …before an aseptic task (e.g. vessel catheter procedure, change of dressing, infusion preparation), (in %): __%/ …after body fluid exposure, (in %): __%/ …after patient contact, (in %): __%/ …after contact with patient surroundings, (in %): __%.

In computing the *WHO-5-based overall compliance rate*, the overall rate extrapolated from the WHO-5 indications-specific rates, the distribution of these indications was taken into account. That is, the relative frequency of each indication’s occurrence in studies reporting hand hygiene opportunities based on the WHO-5 were used as weights. These weights were determined by a review conducted by the first author. Following taxonomies of review types [29–31], this represents a *systematized review* of clinical epidemiological studies that include information on absolute or relative frequencies of hygienic hand disinfection opportunities assessed by observation for all five WHO-5 indications. Details of this review are given in the “Additional file 1” document, which follows the Preferred Reporting Items for Systematic reviews and Meta-Analyses (PRISMA) statement [32] as applicable to the systematized review format.

#### Observed hand hygiene compliance

Hand hygiene compliance rates assessed via direct observation were determined in the participating hospital clinics from October 2018 to August 2020 according to the WHO standard [33]. Items were embedded in an observation sheet for individual patient visits during ward rounds; the sheet included a total of 49 items assessing information on the patient and on SSI-preventive interventions. For each individual hand hygiene opportunity, the WHO-5 indication, as well as the sex and occupation in terms of profession (i.e., physician vs. nurse) of the health care worker observed was recorded. Observations were performed by the IPC teams of the participating hospitals, and the individuals on these teams had been trained and could seek supervision in the use of the observation sheet by the WACH team. The observations were conducted overtly but without intervening in the process of care, i.e., with a definite diagnostic focus.

### Statistical analysis

After a description of the questionnaire survey sample by sociodemographic variables, mean self-reported and directly observed hand hygiene compliance rates were calculated for physicians and nurses. The rates of compliance of these two groups for every WHO-5 indication and their overall rates of compliance were then compared via t-tests (self-reports) or Chi^2^-tests (observations). To test the differences between the single-item overall self-reported compliance rate and the rate extrapolated from the WHO-5 items, t-tests for paired samples were employed. Finally, as an indicator of individual overestimation, explorative multiple linear regression analyses of the differences between these two overall self-report measurements were conducted for physicians and nurses to identify sociodemographic and occupational correlates. When there were significant differences between subgroups, additional stratified analyses were conducted. For all statistical analyses, the software package IBM© SPSS© v27 was used.

## Results

### Sample description

Overall, 336 of 1.068 eligible health care workers completed the questionnaire (response rate: 31.5%; reasons for nonparticipation could not be assessed). Of these, 18 were excluded from the analysis due to a missing value in the occupational group variable, since stratification as “physician” and “nurse” was seen as key to the aims of the study. Thus, the analysis sample included data from N = 318 workers (29.8% of those eligible), 93 of whom physicians (response rate: 28.4%) and 225 of whom were nurses (response rate: 30.4%).

Table 1 provides an overview of the sociodemographic and occupational characteristics of the respondents in the sample. Women were in the majority among nurses (82%), while among physicians, the distribution was less skewed (54.3% men). Age showed fewer differences; approximately half of the respondents in each subsample were 40 years of age or younger (physicians: 48.4%, nurses: 51.6%). Approximately one-third (31.2%) of the participating physicians and one-fifth (20%) of the nurses were members of the local anesthesiology team, while all others worked in one or both of the surgical departments. Regarding specialty, notably all ward nurses were affiliated with a surgical department.

**Table 1:** Description of WACH questionnaire survey sample by sex, age, department, and occupational group^#^

		Physicians N = 93	Nurses N = 225
Sex			
Women	N (%)	42 (45.7%)	178 (82.0%)
Men	N (%)	50 (54.3%)	39 (18.0%)
Age (in years)			
< 18	N (%)	0 (0.0%)	3 (1.4%)
18–30	N (%)	14 (15.4%)	53 (24.7%)
31–40	N (%)	30 (33.0%)	55 (25.6%)
41–50	N (%)	23 (25.3%)	47 (21.9%)
51–60	N (%)	18 (19.8%)	49 (22.8%)
> 60	N (%)	6 (6.6%)	8 (3.7%)
Department			
Orthopedics/Trauma surgery	N (%)	38 (40.9%)	75 (33.3%)
General/Visceral surgery	N (%)	21 (22.6%)	58 (25.8%)
Both surgical departments	N (%)	5 (5.4%)	47 (20.9%)
Anesthesiology	N (%)	29 (31.2%)	45 (20.0%)
Specialty			
Surgical specialists	N (%)	42 (45.2%)	–
Surgical residents	N (%)	20 (21.5%)	–
Anesthesiology specialists	N (%)	15 (16.1%)	–
Anesthesiology residents	N (%)	14 (15.1%)	–
Physician assistants	N (%)	2 (2.2%)	–
Ward nurses	N (%)	–	102 (45.3%)
Perioperative nurses	N (%)	–	78 (34.7%)
Anesthesiology nurses	N (%)	–	45 (20.0%)
Occupational group			
Surgeons	N (%)	64 (68.8%)	–
Anesthesiologists	N (%)	29 (31.2%)	–
Surgical nurses	N (%)	–	180 (80.0%)
Anesthesiology nurses	N (%)	–	45 (20.0%)

^#^Any data not adding up to the relevant total due to missing values

### Hand hygiene compliance by WHO-5 indications

**Fig. 1:**
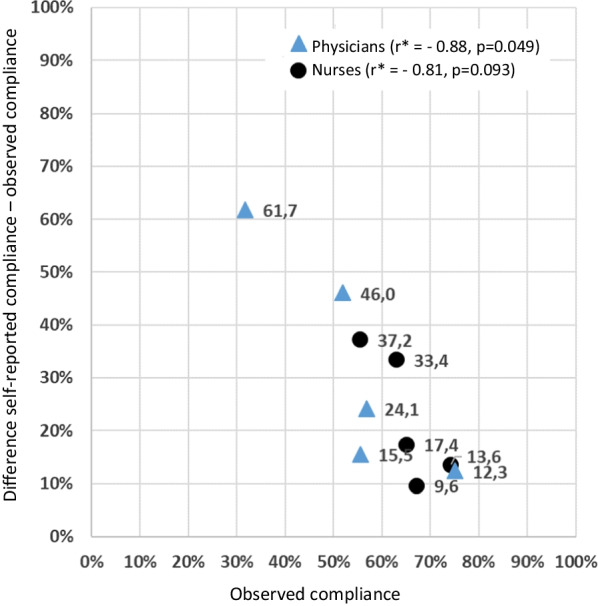
Differences between self-reported and observed WHO-5-specific hand hygiene compliance rates by level of observed compliance^$^. Notes: ^$^For more details, see Table 2. *Pearson correlation coefficient (overall r = −0.87, *p* = 0.001)

As Table 2 shows, no significant mean differences in self-reported compliance between physicians and nurses were found (except for “after body fluid exposure”); the estimates were on a high level (all above 80% except for “after contact with patient surroundings”). In contrast, compliance rates based on observations during ward rounds in the participating hospitals were significantly higher among nurses than among physicians, except for “after patient contact”. Additionally, these estimates were numerically lower than the self-reports, with no percentage (except for “after patient contact”) exceeding 70%. Notably, overestimation in terms of the percentage differences between self-reported and observed rates, which across the WHO-5 ranged from 15.5% (physicians) and 9.6% (nurses) for “after contact with patient surrounding” to 61.7% (physicians) and 37.2% (nurses) for “before an aseptic task”, showed numerically strong negative correlations with the observed rates (see Fig. 1). The supplementarily calculated Spearman’s rho was −0.90, *p* = 0.037, for both physicians and nurses. The correlations of the difference between self-reported and observed compliance divided by the highest possible overestimation as depending on the level of observed compliance were r = −0.63, *p* = 0.26, and rho = −0.60, *p* = 0.285 for physicians and r = −0.61, *p* = 0.28, and rho = −0.60, *p* = 0.285, for nurses (not shown).

**Table 2:** (a) Self-reported and (b) observed hand hygiene compliance among physicians and nurses by WHO-5 indications

		(a) Self-reported compliance			(b) Directly observed compliance^#^		
		Physicians (N = 93)	Nurses (N = 225)	*p*^µ^	Physicians (N = 2421)	Nurses (N = 971)	*p*^µ^
“before patient contact”							
(0–100)	N^§,$^	92	218		902	294	
	(a) Mean Rate (b) Rate	81.0%	82.4%	0.522	56.9%	65.0%	0.014
	95%-CI	77.0%|85.0%	80.2%|84.6%		53.6%|60.1%	59.5%|70.5%	
“before an aseptic task”							
(0–100)	N^§,$^	90	206		246	155	
	(a) Mean Rate (b) Rate	93.4%	92.7%	0.634	31.7%	55.5%	< 0.001
	95%-CI	90.7%|96.1%	91.3%|94.2%		25.9%|37.6%	47.6%|63.4%	
“after body fluid exposure”							
(0–100)	N^§,$^	93	215		229	135	
	(a) Mean Rate (b) Rate	98.0%	96.4%	0.028	52.0%	63.0%	0.041
	95%-CI	97.1%|98.9%	95.3%|97.5%		45.4%|58.5%	54.7%|71.2%	
“after patient contact”							
(0–100)	N^§,$^	93	218		722	256	
	(a) Mean Rate (b) Rate	87.5%	87.8%	0.875	75.2%	74.2%	0.754
	95%-CI	84.2%|90.7%	85.8%|89.7%		72.1%|78.4%	68.8%|79.6%	
“after contact with patient surroundings”							
(0–100)	N^§,$^	93	214		322	131	
	(a) Mean Rate (b) Rate	71.1%	76.8%	0.051	55.6%	67.2%	0.023
	95%-CI	66.1%|76.2%	74.1%|79.5%		50.1%|61.0%	59.0%|75.3%	

^#^Direct observations during ward rounds (N relate to opportunities)

^§^Any totals not adding up to column headers due to missing values

^$^Individuals for self-reported and opportunities for observed compliance

^**µ**^*p*-value for comparison between physicians and nurses

### Overall hand hygiene compliance

Table 3 shows the distribution indices of the focal variables in the present analysis, i.e., the self-reported overall compliance in terms of the single-item rate, and the extrapolated rate based on the WHO-5. Mean compliance rates exceed 80% without exception, and no significant differences between physicians and nurses were found in the mean values, the median values or the standard deviations. In contrast, the mean difference between the single item rate and the WHO-5-based rate was higher among physicians than among nurses (+ 3.1% vs. −1.1%) and statistically significant (*p* = 0.022 vs. *p* = 0.099 for the median values).

**Table 3:** Self-reported overall hand hygiene compliance based on (a) single item and (b) WHO-5-items ((c) = difference)

Variable	Range		Physicians (N = 93)	Nurses (N = 225)	*p*^µ^
(a) Single item rate	(0 to 100)	N^§^	85	141	
		Mean Rate	87.2%	84.4%	0.197
		95%-CI	84.3%|90.0%	81.5%|87.2%	
		Median	90%	90%	0.372’
		Mode	90%	80%	
		Std. Dev	13.4	17.0	0.110
		IQR	80%|100%	80%|99%	
		Skewness	−1.6	−1.9	
		Kurtosis	3.4	4.9	
(b) WHO-5-based rate^~^	(0 to 100)	N^§^	85	141	
		Mean Rate	84.1%	85.5%	0.461
		95%-CI	81.3%|87.0%	83.3%|87.7%	
		Median	85%	89%	0.296’
		Mode	100%	100%	
		Std. Dev	13.0	13.5	0.881
		IQR	77%|94%	79%|95%	
		Skewness	−1.3	−1.7	
		Kurtosis	2.3	4.3	
(c) Difference (a)–(b)	(−100 to 100)	N^§^	85	141	
		Mean Rate	3.1%	−1.1%	0.022
		95%-CI	0.2%|5.8%	−3.3%|1.0%	
		Median	1%	0%	0.099’
		Mode	0%	0%	
		Std. Dev	13.4	12.8	0.910
		IQR	−3.2%|7.1%	−8.3%|5.8	
		Skewness	1.5	−0.6	
		Kurtosis	6.7	2.6	

^§^Any totals not adding up to column header due to missing values

^**µ**^*p*-value for comparison between physicians and nurses

^~^Calculated based on the mean proportions shown in Table A1 (see Additional file 1-document) as weights

’Mann–Whitney-U-Test

**Fig. 2:**
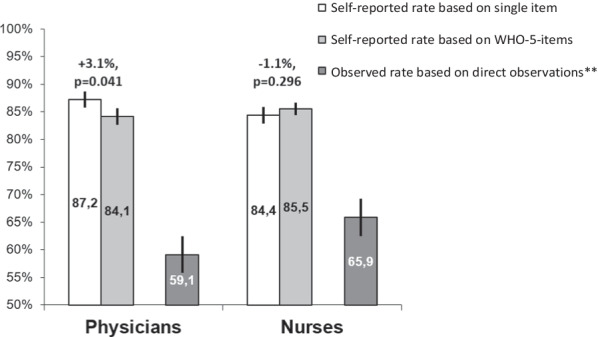
Overall hand hygiene compliance among physicians and nurses by different operationalizations in the WACH-study*. Note: *Whiskers indicate 95%-confidence intervals. **Comparison between physicians and nurses: *p* < 0.001

Figure 2 shows the pattern of mean self-reported overall compliance rates and highlights both the intraindividual differences between the single-item and the WHO-5-based operationalizations and the comparison with directly observed overall compliance in the participating hospitals. The observed overall compliance of nurses exceeded that of physicians by 6.8% (p < 0.001). Consistent with the indication-specific results in Table 2, the self-reported estimates of compliance are numerically higher than the rates of compliance measured by observation. The numerical differences were greater in the case of lower observed compliance (physicians: single-item rate 28.1%, WHO-5-based rate 25%; nurses: 18.5% and 19.6%, respectively).

The central hypothesis that the single-item overall compliance assessment would result in a higher estimate of compliance than the WHO-5-based measurement was true for physicians but not for nurses. That is, physicians provided a mean compliance estimate of 87.2% using the single-item measure, and that estimate was 3.1% higher than the estimate based on the WHO-5 indication-specific items (*p* = 0.041).

#### Explorative analysis of correlates of the single-item overestimation effect among physicians

**Fig. 3:**
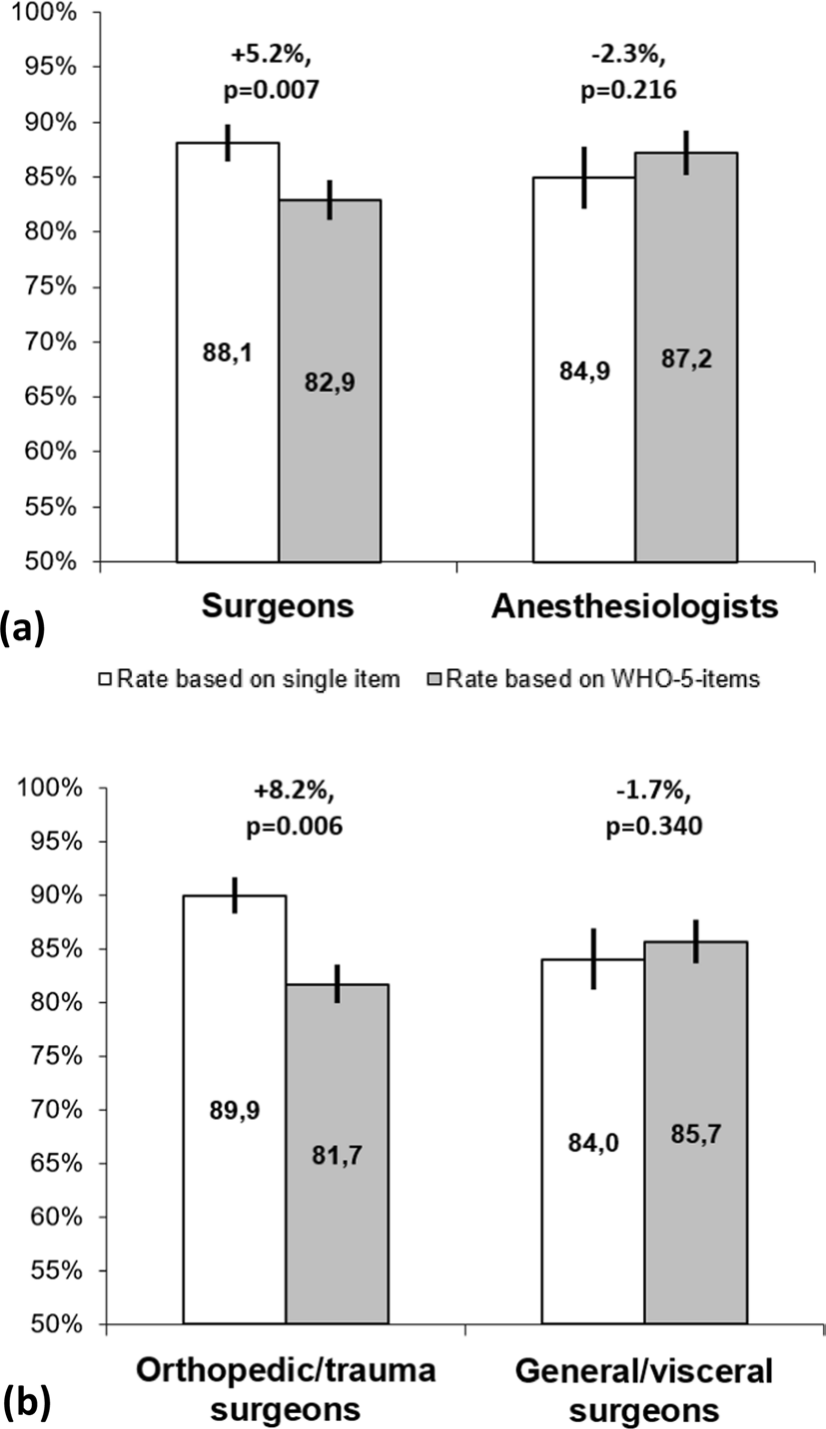
Difference between self-reported hand hygiene compliance based on single item versus WHO-5-items among physician subgroups*. Note: *In % with 95%-confidence intervals

Due to evidence of especially low hand hygiene compliance among orthopedic surgeons [34], explorative analyses were conducted at the individual level for the single-item overestimation effect among physicians. First, neither sex nor age were associated with a difference between the single-item and the WHO-5-based overall compliance rate, but specialty was (see Table 4(a)). As Fig. 3a shows, this effect was limited to surgeons. Finally, among surgeons, the effect only emerged among the participants whose specialty was orthopedics/trauma surgery; there was no evidence of such an effect among the participants who specialized in general/visceral surgery (see Table 4(b) and Fig. 3b).

**Table 4:** Multiple linear regression analyses on difference between single item- and WHO-5-based self-reported overall compliance (physicians and surgeons)

	(a) Physicians						(b) Surgeons				
	Unstandardized coefficients		Standardized coefficients	t	*p*		Unstandardized coefficients		Standardized coefficients	t	*p*
	B	SE	β				B	SE	β		
Intercept	−2.68	6.62		−0.40	0.687	Intercept	1.61	9.00		0.18	0.859
Sex (women: 0, men: 1)	2.18	2.91	0.08	0.75	0.456	Sex (women: 0, men: 1)	3.29	3.94	0.11	0.84	0.408
Age (≤ 40: 0, > 40: 1)	−2.09	2.89	−0.08	−0.72	0.472	Age (≤ 40: 0, > 40: 1)	−5.43	3.97	−0.19	−1.37	0.178
Specialty (anesthesiology: 0, surgery: 1)	7.58	3.15	0.26	2.40	**0.019**	Department (general/visceral: 0, orthopaedics/trauma: 1)	8.91	4.18	0.29	2.13	**0.038**

## Discussion

The results of this study can be summarized as follows. First, significant differences between physicians and nurses in hand hygiene compliance were found by direct observation (both overall and for all WHO-5 indications except “after patient contact”) and were in favor of nurses, while no such differences were found for self-reported compliance (except for “after body fluid exposure” in favor of physicians). With respect to our first hypothesis, overestimation in terms of the numerical difference between self-reported and observed compliance, correlated negatively with observed compliance levels across the WHO-5 indications, particularly among physicians. With respect to the second and main hypothesis of this paper, self-reported overall compliance was higher when it was assessed using a single item than when it was assessed using the relative frequency-weighted index of the WHO-5 items, again among physicians. This was not the case among nurses. In explorative analysis among physicians, this effect was confined to surgeons (vs. anesthesiologists) and specifically to orthopedic or trauma surgeons (vs. physicians who perform general/visceral surgery).

Before the results are discussed further, the limitations of this study must be considered. First, the questionnaire survey response rate was 30% overall. This is lower than the global average response rate of 53% reported for surgical doctors [35]). However, indirect approaches such as that used in the present survey (i.e., distributing a written questionnaire from the WACH team to the survey respondents via the in-house IPC-teams) were not reviewed in [34]. It is probable that the lack of monetary or similar incentives, lack of personalization of the questionnaire, and the relative length of the questionnaire [36] affected the response rate in the WACH-survey. Additionally, the response rate of 73% among surgical doctors in orthopedics in the WACH questionnaire pretest [37] was achieved in a university hospital clinic—a context in which individual health care workers may have a higher affinity for research, and in which there was extraordinary support for the survey by the clinic’s medical director. However, while efforts should be made to increase response rates in future studies of self-overestimation of compliance, there is also a decreasing trend of response rates among surgical doctors [35], and the response rate in the present survey at least falls within one standard deviation of the average rate reported in 2019.

Second, while all self-reported compliance rates were assessed prior to the start of the COVID-19 pandemic, 21% of the hand hygiene opportunities were observed after its onset in March to July 2020. This is relevant because hand hygiene compliance has been reported to have increased among both physicians and nurses during the COVID-19 pandemic [38]. Indeed, when opportunities observed after the onset of the pandemic are omitted, the compliance rates observed in this study decrease to 58.6% (95%-CI: 56.4–60.7%) and 61.1% (57.1–64.9%) for physicians and nurses, respectively. However, this in fact increases the difference between the self-reported and the observed compliance rates, while at the same time, the correlations of the WHO-5-specific differences did not change (r = 0.86, *p* = 0.001 overall, and 0.86, *p* = 0.064, and 0.87, *p* = 0.058 for physicians and nurses, respectively). Thus, the hypotheses of this study are largely unaffected by the effects of the COVID-19 pandemic. Results when observed compliance is restricted to opportunities from the trial’s baseline assessments also speaks for this assertion (for preliminary trial results, see [39]).

Third, the size of most of the significant differences and associations was small. One reason for this may be that the single item and the WHO-5 items were combined in the same questionnaire, and respondents may have been influenced in their answers to the second assessment because they had already thought about their hand hygiene compliance. However, while the eta-squared statistic for the focal paired samples t-test of the two self-reported overall compliance assessments among physicians was small (0.049) [40]), in the explorative analysis of orthopedic/trauma surgeons it was 0.208, and thus considerably larger. Also, even the 3.1% difference reported in Fig. 2 lies within a range indicative of different levels of overconfidence described in studies on the Dunning-Kruger effect, i.e. the phenomenon that people unskilled in a specific task do not have enough expertise to see their own limitations [41, 42]. At the same time, the comparisons between the self-reported and the observed compliance rates were restricted in methodical terms due to the differences in the data sources, i.e., with individuals as the unit of observations in the former case and with hand hygiene opportunities as the unit of observations in the latter case. Aside from some early studies in which there was the opportunity to link questionnaires with hand hygiene observations at the level of individuals [43–45], the absence of self-reports and observational data from the same sample of health care workers has become a common problem; this is probably due to data and/or employment protection considerations and regulations. That is, one cannot be sure whether and to what extent the direct observations represent the true compliance of participants in a survey on self-reported compliance, even if, like in the present study, observations and self-reports come from the same study population of health care workers. Additionally, effect size measures are not readily available in such cases, despite the existence of numerically large differences. All told, analyses in which more determinants of hand hygiene compliance, in terms of self-reported and observed assessments, are used are warranted to increase explained variability.

Finally, for practical and managerial reasons within the WACH project, the directly observed compliance rates used for comparison purposes were determined only during ward rounds. This may to some extent compromise comparisons with the self-reports because the latter were elicited without any confinement to particular workflows or settings. Additionally, this was also the reason we used the relative frequencies of the WHO-5 indications from the systematized review rather than from the observed compliance data collected in WACH, i.e., so that the distribution of the opportunities across the indications would be based on a broader and more representative database (it can be noted that the present results did not change considerably when the WACH-data were utilized). However, as the test of our main hypothesis was not dependent on the directly observed data, we opted to use the relative frequencies from the systematized review as the basis for obtaining the compliance estimates.

With these limitations in mind, it seems safe to say that the present study is the first to analyze the role of item difficulty in the overestimation of hand hygiene compliance among health care professionals, in this case physicians and nurses in the surgical context. Besides comparably larger overestimation, higher self-reported overall compliance based on the single-item measure as opposed to the WHO-5 items (this being our main hypothesis derived from overconfidence theory) was found in physicians. While this is consistent with the commonly lower compliance of physicians compared with that of nurses [16, 21–24], it is psychologically even more intriguing that this finding fits with evidence that physicians judge their hand hygiene guideline knowledge less favorably than do nurses [46]. Specifically, “…as participants less skilled in a task can show even greater overestimation than their peers, perhaps due to a lack of awareness regarding what they do and do not know…” [1, p. 3), it is plausible at least that not only motivational, but also cognitive factors play a role in this context. In fact, a recent study showed that (over)confidence did not correlate with social desirability, but rather represented a knowledge factor [47]. While that study involved university students in different disciplines rather than health care workers, it does support the importance of further research to elucidate whether physicians’ overestimation of their hand hygiene is truly a question of motivated social desirability and impression management, or not, more simply, an indication that they may not be aware of what they do in this regard. The fact that explorative analyses revealed that the contrast between the single-item-based results and the WHO-5-based results was confined to orthopedic/trauma surgeons is consistent with earlier research [34], but further scrutiny regarding differences related to physicians’ specialties is needed.

At the same time, as found in our comparison of self-reported and observed compliance, overestimation of hand hygiene also occurs among nurses. Even the negative correlation between observed compliance levels (as a proxy for task difficulty) and the degree of overestimation across the WHO-5 among nurse was comparable to that among physicians. However, the contrast between the single-item and the WHO-5-based self-reported overall compliance measurements did not show here. In fact, the contrast was reversed: for nurses, the results show numerically higher self-reported compliance based on the WHO-5 index, a finding which is intriguing. It is possible that nurses use their higher awareness of hand hygiene guidelines to (implicitly) calibrate their response to the single item by a “discount” based on a notion of some kind of “empirical realism” regarding their compliance.

Further implications of the present study include the following. For research, future studies should assess relative frequencies of the WHO-5 indications not only for observed hand hygiene, but for self-reported hand hygiene as well. That is, considering health care workers’ individually perceived distribution of the WHO-5 indications may further clarify the psychology of overestimation by comparing them to average relative frequencies such as those determined on the basis of observations in our systematized review (see “Additional file 1” document). Additionally, this study analyzed overestimation but not the other two “faces of overconfidence” [1]: overplacement (i.e., exaggerated beliefs that one is better than others) and overprecision (i.e., excessive certainty that one’s beliefs are accurate). Since these three forms of overconfidence are interrelated but manifest under different conditions (for instance, the opposite association between overplacement and task difficulty predicted from theory), are caused by different factors, and are affected by different consequences, simultaneous analyses of all three facets seem promising.

For practice, implications may be delineated as follows. Given overestimation is a strong psychological barrier to compliance and slightly stronger for the overall self-assessment measure based on the single item, promoting realistic views of their hand hygiene compliance may profit from explicit reference to the WHO-5. In this context, it is noteworthy that observed compliance is highly correlated with overestimation across the WHO-5 indications (see Fig. 1), but that there is also converging evidence that the moments “before an aseptic task” and “after body fluid exposure” are affected by the highest overestimates (see Table 2 and [17, 19, 20]). That is, interventions to reduce overestimation are more on target if they are implemented in a WHO-5-specific manner from a cognitive psychology perspective as well, since more precise beliefs tend to reduce overestimation [13]. While this presupposes WHO-5-specific data, which is not trivial regarding costs, it stands as a prerequisite for immediate personalized feedback and individualized action planning [48].

## Conclusions

For hand hygiene compliance promotion overestimation of hand hygiene compliance by health care professionals and the resulting low and sometimes virtually absent validity of such self-reports [45, 49, 50]) does not make these self-assessments irrelevant. On the contrary, overestimation biases may be strong psychological barriers to change, in that people with this mindset will be “…likely to be oblivious to current campaigns” [45, p. 421]. It is therefore important to recognize these barriers. To overcome them, behavior change techniques [51] may be used. The inconsistency between self-reported overall compliance based on the single item versus the WHO-5-items found in the present study among physicians (specifically orthopedic/trauma surgeons) may add yet another facet to this approach. In our view, the aim is that hand hygiene compliance self-assessments become realistic, especially because this is a prerequisite for effective action control of hand hygiene as an ongoing, practically never-ending everyday task [52–56].

## Supplementary Information

**Additional file 1**. Distribution of hand hygiene opportunities according to the “5 Moments of Hand Hygiene” (WHO-5) in highly developed countries: A systematized review

## Data Availability

The data analyzed in this paper are available from the corresponding author upon reasonable request. This manuscript complies with the STrengthening the Reporting of OBservational studies in Epidemiology (STROBE) Statement for cross-sectional studies (https://www.strobe-statement.org/index.php?id=strobe-home).
