# Supplementary material for: Do task and item difficulty affect overestimation of one’s hand hygiene compliance? A cross-sectional survey of physicians and nurses in surgical clinics of six hospitals in Germany
Source: Antimicrob Resist Infect Control. 2022 Dec 2;11:147. doi: 10.1186/s13756-022-01188-7 (PMC9716516; doi:10.1186/s13756-022-01188-7)
Supplement: Supplementary file 1 — Additional file 1. Distribution of hand hygiene opportunities according to the “5 Moments of Hand Hygiene” (WHO-5) in highly developed countries: A systematized review [file 13756_2022_1188_MOESM1_ESM.pdf]

Supplementary Material

Distribution of hand hygiene opportunities according to the "5 Moments of Hand Hygiene" (WHO-5) in highly developed countries: A systematized review

1. Title

Distribution of hand hygiene opportunities according to the "5 Moments of Hand Hygiene" (WHO-5) in highly developed countries: A systematized review

2. Abstract

This review aimed to provide an estimate of the mean frequency distribution of hand hygiene opportunities in highly developed countries according to the indications defined by the World Health Organization (WHO-5). PubMed database was searched through 11 May 2021 for original studies reporting absolute or relative frequencies of hand hygiene opportunities according to the WHO-5 classification. One reviewer (first author) screened the records, selected the studies and collected data from them to conduct a systematized review. The review includes N=23 studies from 13 countries with a total of 1,959,358 opportunities (mean: 85,189; interquartile range: 659-16,505). The mean proportions of the indications "before patient contact", "before an aseptic task", "after body fluid exposure", "after patient contact", and "after contact with patient surroundings" were 30.0% (95% confidence interval: 26.5%|33.6%), 10.7% (7.7%|13.8%), 10.6% (8.7%|12.4%), 31.2% (27.3%|35.1%), and 17.4% (13.4%|21.4%), respectively. Despite limitations (among others, the use of a single reviewer and a single database only, and variable study size), this – and to our knowledge the first – quantitative review of the distribution of hand hygiene opportunities across WHO-5 indications provides plausible and robust estimates of the relative frequencies of the indications. No funding was received for this review, which was conducted in the context of a doctoral student assignment to provide WHO-5 weights for the analyses in the manuscript to which it is appended. Thus, no registration to a registry has been filed.

3. Rationale

Assessment and feedback of health care workers' hand hygiene compliance (defined as the ratio of disinfections to opportunities for disinfection) remains a cornerstone of infection prevention and control (IPC). However, there are few data on the expected number of opportunities per "5 Moments of Hand Hygiene", i.e., the indications defined by the World Health Organization (2009) (WHO-5). An estimate

of the mean frequency distribution of these indications may be used as a denominator in calculating compliance rates when only the numerator, i.e., the number of disinfections, is known, or as weights for calculating overall compliance when only WHO-5-specific compliance rates are available.

4. Objective

To provide an estimate of the mean frequency distribution of hand hygiene opportunities by indications (WHO-5) in highly developed countries.

5. Eligibility criteria

The inclusion criteria were:

- original study;
- conducted in one or more highly developed countries (human development index [HDI]) >= 0.75);
- explicit use of the concept of hand hygiene opportunities;
- reporting the absolute or relative frequencies of hand hygiene opportunities for all WHO-5 indications (before patient contact, before an aseptic task, after body fluid exposure, after patient contact, and after contact with patient surroundings);
- use of direct observation (Sax et al., 2009; WHO, 2009), video or electronic observation.

Studies that distinguished more than the five indications were excluded.

6. Information sources

The PubMed database (https://pubmed.ncbi.nlm.nih.gov) was searched through 11 May 2021.

7. Search strategy

“Hand hygiene opportunities” was searched, using neither quotation marks nor filters or limits.

8. Selection process

Only one reviewer (first author) screened the records (thus rendering this review to qualify as a systematized review; Grant & Booth, 2009; Sutton et al., 2019). No automation tools were used. Titles and abstracts were screened based on the inclusion and exclusion criteria. If an abstract clearly indicated that no absolute or relative frequencies of hand hygiene opportunities in terms of the WHO-5 indications were reported in the study, the record was excluded. Additionally, studies conducted from countries with an HDI < 0.75 were also excluded.

9. Data collection process

Only one reviewer (first author) collected data from the included studies (reflecting the systematized review classification). Only data on absolute or relative frequencies of hand

hygiene opportunities in terms of the WHO-5 indications were extracted. No data were obtained or confirmed through communication with study investigators, and no automation tools were used in the process.

10. Data items

Data on the absolute or relative frequencies of hand hygiene opportunities in terms of the WHO-5 indications were sought. If studies reported such data for different points in time or contexts (e.g., before and after an intervention, or in different wards), the opportunities were summed. If studies reported the overall number of observed opportunities together with the distribution of the WHO-5 as percentages, the absolute numbers of opportunities per WHO-5 indication were calculated by multiplying the overall number with these percentages to obtain the number per indication.

11. Study risk of bias assessment

The study settings, designs, and methods employed for hand hygiene observations (direct or video observation) were coded. Due to the austere objective of determining the empirical distribution of hand hygiene opportunities across the WHO-5, systematic assessment of risk of bias was not conducted.

12. Effect measures

The relative frequencies of hand hygiene opportunities classified according to the WHO-5 indications were averaged. In doing so, each study was weighted equally, since weighting by study size (i.e., by the number of hand hygiene opportunities observed) would have introduced a bias in that size does reflect the resources available for the studies rather than real-world proportions of the numbers of opportunities.

13. Synthesis methods

Only one tabular synthesis was conducted, and all included studies were eligible. No methods for handling missing summary statistics or data conversions were used. The methods used to tabulate the results of individual studies and syntheses were confined to the calculation of simple absolute and relative frequencies. The heterogeneity of the study results was described by providing 95% confidence intervals. Neither methods to explore causes of heterogeneity nor sensitivity analyses were used.

14. Reporting bias assessment

No methods were used to assess the risk of bias due to missing results in the synthesis arising from reporting biases.

15. Certainty assessment

No methods to assess certainty or confidence in the body of evidence for the outcome were used.

16. Study selection

Figure A1 shows the study selection process. Reports were not sought. Of the initial 529 records, no study was removed before screening, and 454 studies were excluded after screening. Of the 75 remaining studies, 39 reported no distribution of WHO-5 hand hygiene opportunities, 10 were from countries with an HDI < 0.75, two reported fewer than five hand hygiene indications (Goodliffe et al., 2014; Muller et al., 2015), and one study more than five indications (McDonald et al., 2021).

17. Study characteristics

The included studies were conducted in 13 countries (USA 6 studies, Germany 5, Australia 3, Brazil 2, UK 2, China 1, The Netherlands 1, Spain 1, Switzerland 1, and one study was conducted in Australia, Denmark, Finland, and Sweden). There were a total of 1,959,358 hand hygiene opportunities (mean: 85,189, interquartile range: 659-16,505; Germany 1,630,018, Australia 284,659, USA 25,222 Switzerland 12,085, Brazil 4,466, UK 974, Spain 456, Sweden 418, Denmark 425, The Netherlands 285, Finland 237, and China 113). Field times ranged from 2008 to 2020.

18. Risk of bias in studies

Twenty of the studies were cross-sectional observational studies; one study had a before-after design, one was a quasi-experimental interventional study, and one was a cluster-randomized control trial, Additionally, 20 studies used direct hand hygiene observations, two studies used video recording, and one study used electronic technology. In accordance with section 11., no systematic assessments of risk of bias are reported.

19. Results of individual studies

Table A1 shows the results of the 23 studies. The standard deviation of the percentages across the indications ranged from 4.1 (Vikke et al., 2019) to 21.9 (Randle et al., 2013); the average was 12.3.

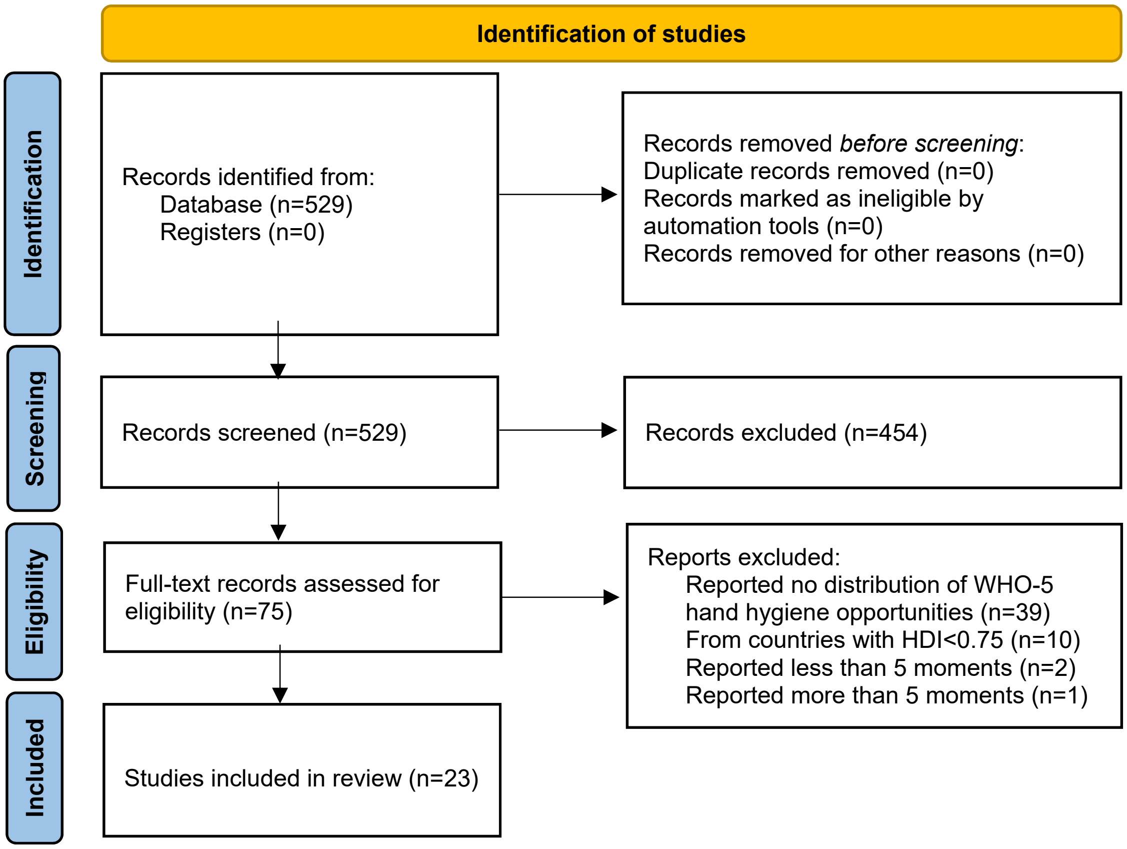

Figure A1: Flow diagram of the study selection process (following Page et al., 2021)

20. Results of synthesis

The mean percentages of the indications “before patient contact”, “before an aseptic task”, “after body fluid exposure”, “after patient contact”, and “after contact with patient surroundings” were 30.0% (95% confidence interval: 26.5%|33.6%), 10.7% (7.7%|13.8%), 10.6% (8.7%|12.4%), 31.2% (27.3%|35.1%), and 17.4% (13.4%|21.4%), respectively. In accordance with section 13., neither the results of the investigations of the possible causes of heterogeneity among the study results nor the results of sensitivity analyses conducted to assess the robustness of the synthesized results are presented.

Table A1: Main characteristics of and hand hygiene opportunities in included studies (N=23)[#]

Publication	Country, Field time, Setting, Study design, Hand hygiene observation method	Before patient contact: $N_{opportunities}$ (proportion)	Before an aseptic task: $N_{opportunities}$ (proportion)	After body fluid exposure: $N_{opportunities}$ (proportion)	After patient contact: $N_{opportunities}$ (proportion)	After contact with patient surroundings: $N_{opportunities}$ (proportion)	Total
Aghdassi et al. (2020)	Germany, 2017-18, university hospital, C-RCT*, direct	5,832 (27.2%)	3,996 (18.7%)	1,644 (7.7%)	6,369 (29.7%)	3,583 (16.7%)	**21,424**
Azim & McLaws (2014)	Australia, 2013, 82 public hospitals, observational, direct	70,248 (28.5%)	19,925 (8.1%)	26,893 (10.9%)	74,142 (30.1%)	55,232 (22.4%)	**246,440**
Azim et al. (2016a)	Australia, 2014-15, university hospital, observational, direct	7,334 (34.2%)	1,765 (8.2%)	2,162 (10.1%)	5,824 (27.2%)	4,365 (20.3%)	**21,450**
Azim et al. (201bb)	Australia, 2013, university hospital, observational, direct	5,533 (33.5%)	1480 (9.0%)	1,803 (10.9%)	4,841 (29.3%)	2,848 (17.3%)	**16,505**
Belela-Anacleto et al. (2019)	Brazil, 2017, university hospital, before and after intervention, direct	373 (25.9%)	248 (17.2%)	237 (16.4%)	323 (22.4%)	260 (18.0%)	**1,441**
Bezerra et al. (2020)	Brazil, 2018, university hospital, observational, direct	959 (31.7%)	544 (18.0%)	523 (17.3%)	681 (22.5%)	318 (10.5%)	**3,025**
Buet et al. (2013)	USA, 2011, pediatric care facilities, observational, direct	334 (38.6%)	13 (1.5%)	119 (13.8%)	215 (24.9%)	184 (21.3%)	**865**
Chang et al. (2016)	USA, 2013, three acute care hospitals, observational, direct	682 (31.8%)	166 (7.7%)	160 (7.5%)	454 (21.2%)	684 (31.9%)	**2,146**
Cheng et al. (2011)	China, 2009-10, acute care hospital, observational, electronic	40 (35.4%)	3 (2.7%)	8 (7.1%)	54 (47.8%)	8 (7.1%)	**113**
Derksen et al. (2020)	Germany, 2020, two university hospitals, observational, direct	75 (28.1%)	39 (14.6%)	25 (9.4%)	91 (34.1%)	37 (13.9%)	**267**
Diller et al. (2014)	USA, 2013, teaching hospital, observational validation study, video surveillance	1,901 (21.1%)	654 (7.3%)	539 (6.0%)	2,111 (23.5%)	3,791 (42.1%)	**8,996**
Fulchini et al. (2018)	Switzerland, 2014, tertiary care hospital, retrospective observational, direct	3,418 (28.3%)	1902 (15.7%)	1,113 (9.2%)	4,141 (34.3%)	1,511 (12.5%)	**12,085**

Table A1 continued

Publication	Country, Field time, Setting, Study design, Hand hygiene observation method	Before patient contact: $N_{opportunities}$ (proportion)	Before an aseptic task: $N_{opportunities}$ (proportion)	After body fluid exposure: $N_{opportunities}$ (proportion)	After patient contact: $N_{opportunities}$ (proportion)	After contact with patient surroundings: $N_{opportunities}$ (proportion)	Total
Haac et al. (2018)	USA, 2015, university hospital, retrospective observational, video	375 (36.3%)	178 (17.2%)	19 (1.8%)	376 (36.4%)	81 (8.3%)	**1,034**
Haas & Larson (2008)	USA, 2006-07, university medical center, quasi-experimental, direct	112 (14.8%)	24 (3.2%)	117 (15.5%)	432 (57.1%)	72 (9.5%)	**757**
Hilt et al. (2020)	Netherlands, 2017, five general practice offices, observational, direct	74 (26.0%)	38 (13.3%)	46 (16.1%)	110 (38.6%)	17 (6.0%)	**285**
Kramer et al. (2020)	Germany, 2015-18, 525 hospitals, observational, direct	353,820 (23.8%)	239,327 (16.1%)	204,886 (13.8%)	395,804 (26.6%)	291,805 (19.6%)	**1,485,622**
Randle et al. (2010)	UK, 2010, teaching hospital, observational, direct	291 (44.2%)	3 (0.5%)	93 (14.1%)	142 (21.5%)	130 (19.7%)	**659**
Randle et al. (2013)	UK, 2010, teaching hospital, observational, direct	155 (49.2%)	0 (0.0%)	4 (1.3%)	114 (36.2%)	42 (13.3%)	**315**
Rodríguez-Villare et al. (2019)	Spain, 2018, university hospital, observational, direct	145 (31.8%)	40 (8.8%)	37 (8.1%)	186 (40.8%)	48 (10.5%)	**456**
Stahmeyer et al. (2017)	Germany, 2014, university hospital, observational, direct	320 (16.9%)	536 (28.3%)	261 (13.8%)	411 (21.7%)	368 (19.4%)	**1,896**
Steed et al. (2011)	USA, 2010, teaching and acute care hospital, observational, direct	2,385 (20.9%)	649 (18.7%)	1,199 (7.7%)	3,193 (29.7%)	3,998 (16.7%)	**11,424**
Vikke et al. (2019)	Finland, Sweden, Australia & Denmark, 2016-17, emergency medical services (multicenter), observational, direct	513 (38.2%)	158 (11.8%)	119 (8.9%)	482 (35.9%)	72 (5.4%)	**1,344**
Wetzker et al. (2016)	Germany, 2014, 109 hospitals, observational, direct	29,988 (24.8%)	16,713 (13.8%)	15,862 (13.1%)	34,417 (28.5%)	23,829 (19.7%)	**120,809**
**Total**		**484,907 (24.7%)**	**288,401 (14.7%)**	**257,849 (13.2%)**	**534,913 (27.3%)**	**393,288 (20.1%)**	**1,959,358**
**Mean Proportion**		**30.0%**	**10.7%**	**10.6%**	**31.2%**	**17.4%**	

[#] Listed in ascending alphabetical order by authors; * cluster-randomized controlled trial

21. Reporting biases

In accordance with section 13., no assessments of risk of bias due to missing results in the synthesis arising from reporting biases are presented.

22. Certainty of evidence

In accordance with section 15., no assessments of certainty or confidence in the body of evidence for the outcome are presented.

23. Discussion

This review has limitations. First, it represents not a systematic, but a systematized review (Grant & Booth, 2009; Sutton et al., 2019) in that because it was performed as a doctoral student assignment, there was only one reviewer (first author), and only one database was used (PubMed). Second, it did not differentiate between professional groups, e.g. physicians, nurses, and allied health professionals. Third, medical specialties were not contrasted. Nonetheless, this review represents, to our knowledge, the first quantitative review of the distribution of hand hygiene opportunities across the indications defined by the WHO (WHO-5). Despite considerable variation in the studies' sizes and contexts, it provides plausible and quite robust estimates of the average relative frequencies of the WHO-5. These estimates were usable as weights for the self-reported WHO-5-specific compliance rates in the study to which this review is appended. Further research should elucidate whether the estimates can be used as denominators in cases in which such data are missing, and more generally whether there are of value in increasing the construct validity of hand hygiene assessments (Neo, 2017).

24. Registration and protocol

Because this review was conducted in the context of a doctoral student assignment to provide weights for the WHO-5 indications in the context of the analyses conducted in the above manuscript, no review protocol was prepared and no registration to a registry has been filed.

25. Support

The review received no financial or nonfinancial support, and no funders or sponsors had any role in the review.

26. Competing interests

The authors declare no competing interests.

27. Availability of data, code, and other materials

The data analyzed in this review are available from the corresponding author upon reasonable request.

References (*: study included in the review)

*Aghdassi, S. J. S., Schröder, C., Lemke, E., Behnke, M., Fliss, P. M., Plotzki, C., Wenk J., Gastmeier, P., & Kramer, T. S. (2020). A multimodal intervention to improve hand hygiene compliance in peripheral wards of a tertiary care university centre: a cluster randomised controlled trial. *Antimicrobial Resistance and Infection Control, 9*, 113. doi: 10.1186/s13756-020-00776-9.

*Azim, S., & McLaws, M.-L. (2014). Doctor, do you have a moment? National Hand Hygiene Initiative compliance in Australian hospitals. *The Medical Journal of Australia, 200*(9), 534-537. doi: 10.5694/mja13.11203.

Azim, S., Juergens, C., Hines, J., & McLaws, M.-L. (2016a). Introducing automated hand hygiene surveillance to an Australian hospital: Mirroring the HOW2 Benchmark Study. *American Journal of Infection Control, 44*(7), 772-776. doi: 10.1016/j.ajic.2016.02.012.

*Azim, S., Juergens, C., & McLaws, M.-L. (2016b). An average hand hygiene day for nurses and physicians: The burden is not equal. *American Journal of Infection Control, 44*(7), 777-781. doi: 10.1016/j.ajic.2016.02.006.

*Belela-Anacleto, A. S. C., Kusahara, D. M., Peterlini, M. A. S., & Pedreira, M. L. G. (2019). Hand hygiene compliance and behavioural determinants in a paediatric intensive care unit: An observational study. *Australian Critical Care, 32*(1), 21-27. doi: 10.1016/j.aucc.2018.02.010.

*Bezerra, T. B., Valim, M. D., Bortolini, J., Ribeiro, R. P., Marcon, S. R., & Moura, M. E. B. (2020). Adherence to hand hygiene in critical sectors: Can we go on like this? *Journal of Clinical Nursing, 29*(13-14), 2691-2698. doi: 10.1111/jocn.15293.

*Buet, A., Cohen, B., Marine, M., Scully, F., Alper, P., Simpser, E., Saiman, L., & Larson, E. (2013). Hand hygiene opportunities in pediatric extended care facilities. *Journal of Pediatric Nursing, 28*(1), 72-76. doi: 10.1016/j.pedn.2012.04.010.

*Chang, N.-C. N., Reisinger H. S., Jesson, A. R., Schweizer, M. L., Morgan, D. J., Forrest, G. N., & Perencevich, E. N. (2016). Feasibility of monitoring compliance to the My 5 Moments and entry/exit hand hygiene methods in US hospitals. *American Journal of Infection Control*, *44*(8), 938-940. doi: 10.1016/j.ajic.2016.02.007.

*Cheng, V. C. C., Tai, J. W. M., Ho, S. K. Y., Chan, J. F. W., Hung, K. N., Ho, P. L., & Yuen, K. Y. (2011). Introduction of an electronic monitoring system for monitoring compliance with Moments 1 and 4 of the WHO “My 5 Moments for Hand Hygiene” methodology. *BMC Infectious Diseases, 11*, 151. doi: 10.1186/1471-2334-11-151.

*Derksen, C., Keller, F. M., & Lippke S. (2020). Obstetric healthcare workers' adherence to hand hygiene recommendations during the COVID-19 pandemic: observations and social-cognitive determinants. *Applied Psychology: Health and Well-Being, 12*(4), 1286-1305. doi: 10.1111/aphw.12240.

Diller, T., Kelly, J. W., Blackhurst, D., Steed, C., Boeker, S., & McElveen, D. C. (2014). Estimation of hand hygiene opportunities on an adult medical ward using 24-hourcamera surveillance: validation of the HOW2 Benchmark Study. American Journal of Infection Control, 42*(6), 602-607. doi: 10.1016/j.ajic.2014.02.020.

*Fulchini, R., Kohler, P., Kahlert, C. R., Albrich, W. C., Kuhn, R., Hoffmann, M., & Schlegel, M. (2018). Hand hygiene adherence in relation to influenza season during 6 consecutive years. *American Journal of Infection Control, 46*(11),1311-1314. doi:10.1016/j.ajic.2018.04.203.

Goodliffe, L., Ragan, K., Larocque, M., Borgundvaag, E., Khan, S., Moore, C., McCreight, L., Coleman, B. L., McGeer, A. J., & Mount Sinai Infection Control Team (2014). Rate of healthcare worker-patient interaction and hand hygiene opportunities in an acute care setting. *Infection Control & Hospital Epidemiology*, *35*(3), 225-230. doi: 10.1086/675286

Grant, M. J., & Booth, A. (2009). A typology of reviews: an analysis of 14 review types and associated methodologies. *Health Information & Libraries Journal*, *26*(2), 91-108. doi: 10.1111/j.1471-1842.2009.00848.x.

*Haac, B., Rock, C., Harris, A. D., Pineles, L., Stein, D., Scalea, T., Hu, P., Hagegeorge, G., Liang, S. Y., & Thom, K. A. (2018). Hand hygiene compliance in the setting of trauma resuscitation. *HHS Public Access, 48*(1),165-170. doi:10.1016/j.injury.2016.08.004.

Haas, J. P., & Larson, E. L. (2008). Impact of wearable alcohol gel dispensers on hand hygiene in an emergency department. Academic Emergency Medicine, 15*(4), 3936. doi: 10.1111/j.1553-2712.2008.00045.x.

*Hilt, N., Lokate, M., OldeLoohuis, A., Hulscher, M. E. J. L., Friedrich, A. W., & Voss, A. (2020). Hand hygiene compliance in Dutch general practice offices. *Archives of Public Health, 78*, 79. doi: 10.1186/s13690-020-00464-5.

*Kramer, T. S., Bunte, K., Schröder C., Behnke M., Clausmeyer, J., Reichardt, C., Gastmeier, P., & Walter J. (2020). No increase in compliance before aseptic procedures in German hospitals: a longitudinal study with data from the national surveillance system over four years. *Journal of Hospital Infection, 106*(1), 71-75. doi:10.1016/j.jhin.2020.07.001.

McDonald, M. V., Brickner, C., Russell, D., Dowding, D., Larson, E. L., Trifilio, M., Bick, I. Y., Sridharan, S., Song, J., Adams, V., Woo, K., & Shang, J. (2021). Observation of hand hygiene practices in home health care. *Journal of the American Medical Directors Association*, *22*(5), 1029-1034. doi: 10.1016/j.jamda.2020.07.031.

Muller, M. P., Carter, E., Siddiqui, N. & Larson, E. (2015). Hand hygiene compliance in an emergency department: the effect of crowding. *Academic Emergency Medicine*, *22*(10), 1218-1221. doi: 10.1111/acem.12754.

Neo, J. R. J. (2017). Construct validity-Current issues and recommendations for future hand hygiene research. *American Journal of Infection Control*, *45*(5), 521-527. doi: 10.1016/j.ajic.2017.01.028

Page, M. J., McKenzie, J. E., Bossuyt, P. M., Boutron, I., Hoffmann, T. C., Mulrow, C. D., Shamseer, L., Tetzlaff, J. M., Akl, E. A., Brennan, S. E., Chou, R., Glanville, J., Grimshaw, J. M., Hróbjartsson, A., Lalu, M. M., Li, T., Loder, E. W., Mayo-Wilson, E., McDonald, S., McGuinness, L. A., Stewart, L. A., Thomas, J., Tricco, A. C., Welch, V. A., Whiting, P., & Moher, D. (2021). The PRISMA 2020 statement: an updated guideline for reporting systematic reviews. *BMJ*, *372*, n71. doi: 10.1136/bmj.n71.

*Randle, J., Arthur, A., & Vaughan, N. (2010). Twenty-four-hour observational study of hospital hand hygiene compliance. *Journal of Hospital Infection, 76*(3), 252-255. doi:10.1016/j.jhin.2010.06.027.

*Randle, J., Firth, J., & Vaughan, N. (2013). An observational study of hand hygiene compliance in paediatric wards. *Journal of Clinical Nursing*, *22*(17-18), 2586-2592. doi:10.1111/j.1365-2702.2012.04103.x.

*Rodríguez-Villar, D., Del-Moral-Luque, J. A., San-Román-Montero, J., Gil-de-Miguel, A., Rodríguez-Caravaca, G., & Durán-Poveda, M. (2019). [Hand hygiene compliance with hydroalcoholic solutions in medical students. Cross-sectional study]. *Revista Española de Quimioterapia*, *32*(3), 232-237.

Sax, H., Allegranzi, B., Chraïti, M. N., Boyce, J., Larson, E., & Pittet, D. (2009). The World Health Organization hand hygiene observation method. *American Journal of Infection Control, 37*(10), 827-834. doi: 10.1016/j.ajic.2009.07.003

*Stahmeyer, J. T., Lutze, B., von Lengerke, T., Chaberny, I. F., & Krauth, C. (2017). Hand hygiene in intensive care units: a matter of time. *Journal of Hospital Infection, 95*(4), 338-343. doi: 10.1016/j.jhin.2017.01.011.

*Steed, C., Kelly, W., Blackhurst, D., Boeker, S., Diller, T., Alper, P., & Larson, E. (2011). Hospital hand hygiene opportunities: where and when (HOW2)? The HOW2 Benchmark Study. *American Journal of Infection Control*, *39*(1),19-26. doi:10.1016/j.ajic.2010.10.007.

Sutton, A., Clowes, M., Preston, L., & Booth, A. (2019). Meeting the review family: exploring review types and associated information retrieval requirements. *Health Information & Libraries Journal*, *36*(3), 202-222. doi: 10.1111/hir.12276.

*Vikke, H. S., Vittinghus, S., Giebner, M., Kolmos, H. J., Smith, K., Castrén, M., & Lindström, V. (2019). Compliance with hand hygiene in emergency medical services: an international observational study. *Emergency Medicine Journal, 36*(3), 171-175. doi:10.1136/emermed-2018-207872.

*Wetzker, W., Bunte-Schönberger, K., Walter, J., Pilarski, G., Gastmeier, P., & Reichardt, C. (2016). Compliance with hand hygiene: reference data from the national hand hygiene campaign in Germany. *Journal of Hospital Infection*, *92,* 328-331. doi: 10.1016/j.jhin.2016.01.022.

World Health Organization (WHO) (2009). WHO guidelines of hand hygiene in health care: first global patient safety challenge clean care is safer care. Geneva: WHO. Available at: whqlibdoc.who.int/publications/2009/9789241597906_eng.pdf. Last access: 22 Jan 2022.
